# The lncRNA ADAMTS9-AS2 Regulates RPL22 to Modulate TNBC Progression *via* Controlling the TGF-β Signaling Pathway

**DOI:** 10.3389/fonc.2021.654472

**Published:** 2021-06-09

**Authors:** Kan Ni, Zhiqi Huang, Yichun Zhu, Dandan Xue, Qin Jin, Chunhui Zhang, Changjiang Gu

**Affiliations:** ^1^ Department of General Surgery, Affiliated Hospital of Nantong University, Nantong, China; ^2^ Department of General Surgery, Nantong First people’s hospital, The Second Affiliated Hospital of Nantong University, Nantong, China; ^3^ Department of Pathology, Affiliated Hospital of Nantong University, Nantong, China

**Keywords:** TNBC (Triple negative breast cancer), TGFb (transforming growth factor-beta), lncRNA, ADAMTS9-AS2, signaling pathway

## Abstract

**Background:**

Long non-coding RNAs (lncRNAs) are key regulators of triple-negative breast cancer (TNBC) progression, but further work is needed to fully understand the functional relevance of these non-coding RNAs in this cancer type. Herein, we explored the functional role of the lncRNA ADAMTS9-AS2 in TNBC.

**Methods:**

Next-generation sequencing was conducted to compare the expression of different lncRNAs in TNBC tumor and paracancerous tissues, after which ADAMTS9-AS2differential expression in these tumor tissues was evaluated *via* qPCR. The functional role of this lncRNA was assessed by overexpressing it *in vitro* and *in vivo.* FISH and PCR were used to assess the localization of ADAMTS9-AS2within cells. Downstream targets of ADAMTS9-AS2 signaling were identified *via* RNA pulldown assays and transcriptomic sequencing.

**Results:**

The expression ofADAMTS9-AS2 was decreased in TNBC tumor samples (P < 0.05), with such downregulation being correlated with TNM stage, age, and tumor size. Overexpressing ADAMTS9-AS2 promoted the apoptotic death and cell cycle arrest of tumor cells *in vitro* and inhibited tumor growth *in vivo.* From a mechanistic perspective, ADAMTS9-AS2 was found to control the expression of RPL22 and to thereby modulate TGF-β signaling to control TNBC progression.

**Conclusion:**

ADAMTS9-AS2 controls the expression of RPL22 and thereby regulates TNBC malignancy *via* the TGF-β signaling pathway.

## Introduction

Breast cancer (BC) is a leading cause of cancer-related death in women (1). While many advances in BC diagnosis and treatment have been made in recent years, high rates of tumor chemoresistance and metastasis often result in a poor prognosis (2). There are many molecular subtypes of BC, with triple-negative BC (TNBC) being the most aggressive subtype, accounting for ~20% of BC diagnoses (3). TNBC tumors are so-named owing to their lack of estrogen receptor (ER), progesterone receptor (PR), and human epidermal growth factor receptor 2 (HER2), and these tumors are not susceptible to therapies targeting these receptor signaling pathways (4–6). Further work is needed to improve the 5-year survival rate of BC patients (7), and as such, more work is needed to clarify the molecular mechanisms governing BC progression and recurrence in order to guide appropriate patient treatment.

Approximately 80% of transcripts in cells are non-coding RNAs (ncRNAs) (8), including long ncRNAs (lncRNAs) > 200 nucleotides in length (9). Despite not encoding proteins, lncRNAs are able to control transcription and translation (10–12), thereby regulating the progression of many tumor types including BC (13) by controlling cell survival, proliferation, metastasis, and chemoresistance (14, 15). ADAMTS9-AS2 is a lncRNA encoded on chr3:64684720-64809891 that is differentially expressed in many tumor types (16). When upregulated, ADAMTS9‐AS2 has previously been shown to suppress miR‐223‐3p expression and to thereby control TGFBR3 expression, ultimately inhibiting lung cancer growth (17). In BC, ADAMTS9‐AS2 downregulation is linked to miR-130a-5p upregulation and tamoxifen resistance (18), while in ovarian cancer this lncRNA targets the miR-182-5p/FOXF2 signaling axis to control tumor progression (19). As such, ADAMTS9‐AS2 likely serves as a tumor suppressor in several cancer types, but the mechanistic basis for ADAMTS9-AS2 regulatory activity in TNBC remains to be defined.

Herein, we employed a bioinformatics approach to evaluate differential lncRNA expression profiles in TNBC, and found ADAMTS9-AS2 to be downregulated in this cancer type. We then explored the functional role of this lncRNA in this oncogenic context.

## Materials and Methods

### Clinical Tissues

Sixty-two pairs of TNBC and matched adjacent normal tissue samples were obtained from patients surgically resected at the Affiliated Hospital of Nantong University from January 2018 to July 2020. None of the patients received preoperative chemotherapy or radiotherapy. The tissue samples were stored at -80°C. This study was approved by the Clinical Research Ethics Committee of the Affiliated Hospital of Nantong University.

### Cell Culture

The TNBC cell lines MDA-MB-231 and HCC1937 were acquired from Cell Bank of Type Culture Collection of the Chinese Academy of Sciences (Shanghai, China). MDA-MB-231 and HCC1937 cells were cultured in DMEM (Gibco, Grand Island, NY, USA) and RPMI-1640 (Gibco) media, respectively. Both media were supplemented with 10% fetal bovine serum (GIBCO-BRL, Invitrogen, USA), 100 ug/mL penicillin and 100 U/mL streptomycin (Shanghai Genebase Gen-Tech, Shanghai, China) The cells were maintained at 37°C in a 5% CO2 incubator.

### Real-Time Quantitative Polymerase Chain Reaction (RT-qPCR)

TRIzol reagent (Invitrogen, Carlsbad, CA, USA) was used to isolate total RNA from tissues and cells according to the manufacturer’s instructions. RevertAid First Strand cDNA Synthesis Kit (Thermo Fisher Scientific, USA) was then performed to reverse transcribe total RNA(1 μg) to cDNA. A Roche LightCycler 480 (Roche, Basel, Switzerland) was used to conduct qRT-PCR analyses. Target primers were amplified by SYBR Green Mix (Roche) according to the manufacturer’s instructions. Sequences of the primers Primers used in this study were as follows: ADAMTS9-AS2 (F: 5’-CTACCCCCTCCCAGTCTTCA-3’; R: 5’-GGTCTTGCTCTTTCCTTATCCTCA-3’), RPL22 (F: 5’- GACAACTGAAAGGGGCTACCAAGG-3’; R: 5’- GCACCACAAGGCACCAGAGTC-3’); GADPH (F: 5′-CGCTGAGTACGTCGTGGAGTC-3′; R: 5′-GCTGATGATCTTGAGGCTGTTGTC-3′). All results were calculated and expressed as 2-ΔΔCt. GAPDH was used as endogenous control.

### Cell Transfection

TNBC cells in the logarithmic growth phase were cultured in a 6-well plate (5 × 104 cells/well) for one day. When the cells had settled, the cells were infected with lentivirus with ADAMTS9-AS2 overexpression. The cells were then cultured in the presence of puromycin for 5 days to screen for positively infected cells. When the infection efficiency exceeded 90% under fluorescence microscope and the efficiency was proved by qRT-PCR, the cells were collected for use in experiments. The siRPL22 was transfected into TNBC cells by using Lipofectamine 3000 (Invitrogen). The manufacturer’s instructions were strictly followed.

### Cell Counting Kit-8 (CCK8), Colony Formation and EdU Assay

2000 cells were seeded onto 96-well plates and placed in an incubator with 5% CO2 at 37°C, 10 μl of CCK8 solution was added to each well every 24h. The cell viability was evaluated in a absorbance values of 450 nm. For colony formation assay, 1000 cells were seeded in to 6-well plates for 2 weeks, then fixed in methanol and stained with 0.1% crystal violet. EdU assay (5-ethynyl-20-deoxyuridine) was performed with a commercial kit (Ribobio, Guangzhou, China). The cells in logarithmic growth phase were inoculated in 24 well plates with 5 × 10^4^ cells per well. 200 μL 50 μM EdU medium added to each well for incubation for 2 hours, then the cells were washed 2 times by PBS. 200 μl of 4% paraformaldehyde was added to each well and incubated at room temperature for 30 minutes. 200 μL PBS was used to wash each well. 0.5% TritonX-100 was added to each well and shaked for 10 min. 100 μL of 1× Apollo^®^ Staining reaction solution was added into each well and incubated in dark, with shaking for 30 minutes. 0.5% TritonX-100 was added to each well and shaked for 10 min 2 times. 200 μL methanol was used to wash each well. 100 μL 1 x Hoechst 33342 reaction solution was added to each well and incubated in dark for 30 min. The well was washed by PBS. The rate of proliferating cells was counted by fluorescence microscope. Triplicate is required for each experiment.

### Wound Healing, Migration and Invasion Assays

The transfected TNBC cells were seeded in 6-well plate and scratched with a10 μL pipette tip, then cultured in serum-free medium for 24 h, the width of wounds was examined and normalized to control group. For invasion assays, 5×10^4^ TNBC cells were suspended in serum-free medium and placed into the upper chambers (BD BioCoat, MA, USA) coated with matrigel (BD Biosciences, NJ, USA) in a volume of 200 μL, and then 600 μL 10% FBS medium was added into the bottom chambers. After 24 h, the cells on the upper chambers were removed and cells on the lower compartment were fixed with ethanol and stained by crystal violet, then photographed and counted with microscope. For migration assay, Matrigel was not needed and the left steps were the same as above.

### ADAMTS9-AS2 Subcellular Location Analysis

Living TNBC cell lines were fixed in 4% paraformaldehyde firstly for FISH assay. Then the cells were treated with triton X-100 (Solarbio, China) and subsequently treated with Fluorescent In Situ Hybridization Kit (RiboBio, China) following the manufacturers’ instructions. RNA FISH probes were designed by GenePharma (GenePharma, Shanghai, China). Cytoplasmic and nuclear separation was used by the PARIS Kit (Life Technologies, Carlsbad, CA, USA). 10^7^ living cells were collected and washed by cold PBS. 500 μL of ice-cold Cell Disruption Buffer was added to the cells. Then the cells was lysed by vortex. The lysate was transferred to a tube containing 2X Lysis/Binding Solution for RNA isolation at room temp and then mixed gently. ACS grade 100% ethanol equal was added to the mixture and then the sample mixture was transferred to Filter Cartridge in Collection Tube for centrifugation until all the mixture was through the filter. The flow-through was discarded and the filter was washed by Wash Solution. Elution Solution was preheated to 95°C and added to the filter. the RNA was recovered by centrifugation for 30 sec. qRT-PCR was used to detect the relative expression.

### Tumor Xenografts in Animals

The xenograft mice *in vivo* assays were performed with four-week-old female BALB/c-nude mice (about 18 g), which were purchased from the animal center of Nantong University (Nantong, China). according to the institutional guidelines and approved by the Animal Ethics Committee of Afliated Hospital of Nantong University. The mice were injected with MDA-MB-231 cells (1×107) with LV NC or ADAMTS9-AS2 subcutaneously. The volume of xenograft tumors was measured every 5 days. After 25 days, the mice were executed and tumors were taken out for weighing and subjected to H&E and immunohistochemical staining assay.

### RNA Pulldown Assay

Cell protein was collected from MDA-MB-231. Pierce RNA 3′ End Desthiobiotinylation Kit (Thermo Fisher Scientific, USA) and Pierce Magnetic RNA-Protein PullDown Kit (Thermo Fisher Scientific) were used for RNA pulldown following the manufacturer’s instructions. After Pre-Washing the beads, 50 µl beads was added for the assay. The supernatant was discarded by a magnetic stand and was added an equal volume of 1X RNA Capture Buffer. 50pmol of labeled RNA (GenePharma, Shanghai, China) was added to the beads and incubated for 30 minutes at room temperature with agitation. The supernatant was discarded and washed with 20mM Tris. 100µL of 1X Protein-RNA Binding Buffer (Mixed buffer with collected cell protein) was added to the beads and mixed well. The supernatant was discarded and 100µL of Master Mix was added to the RNA-bound beads for incubating 60 minutes at 4°C with agitation. The mixture was washed with 1X wash buffer twice and the supernatant was discarded. Then 50µL of Elution Buffer was added to the beads and mixed well by vortexing and incubated 30 minutes at 37°C with agitation. Finally the tube was put into a magnetic stand to collect supernatant for downstream analysis. The samples to be tested were identified by mass spectrometry performed by the Shanghai Applied Protein Technology Co, Ltd. Western blot assay was used to prove the downstreamed protein.

### Assays of Production of Lactate and ATP

Lactate Assay Kit II and ATP Colorimetric Assay Kit were used to measure the production of lactate and ATP according to the manufacturer’s instructions (Beyotime, Shanghai, China). Appropriate amount of cells were homogenized in 100 µl corresponding assay buffer provided by the kits. The homogenized cells were centrifuged, and the soluble fraction was analyzed and measured the RLU value with a luminometer (Promega, Madison, WI, USA). ATP levels was estimated based on the standard curve, and normalized to the cell number.

### Western Blot Analysis

The total protein of TNBC cells was exacted with RIPA buffer and separated by sodium dodecyl sulfate polyacrylamide gel electrophoresis, then electransferred onto a PVDF membrane (Bio-Rad, CA, USA). The membranes were blocked with 5% skim milk and incubated with antibodies against Cyclin D1 (1:1000, Proteintech, USA), p27 (1:1000, Proteintech), Bax (1:1000, Proteintech), Bcl-2 (1:1000, Proteintech), E-cadherin (1:2000, Proteintech), Vimentin (1:1000, CST, Danvers, USA), N-cadherin(1:1000, Proteintech), p-ERK1/2 (1:1000, Abcam, Cambridge, UK); ERK1/2 (1:2000, Abcam), Smad2(1:500, Proteintech), Smad7(1:500, Proteintech),TGFBR1(1:500, Proteintech) and GADPH (1:5000, CST) at 4°C overnight and then incubated with secondary antibodies (1:5000, Proteintech) at room temperature for 2 h. Finally, the bands were examined by Immobilob™ Western Chemiluminescent HRP Substrate (Millipore, Billerica, MA, USA) and were detected by Immunoblots visualized by ECL detection system (Quantity One software, BioRad).

### Bioinformatics Analysis

ADAMTS9-AS2 expression levels in a large BC patient cohort were assessed using the Ualcan database (http://ualcan.path.uab.edu) using the search terms “ADAMTS9-AS2,” “Cancer VS. Normal/Cancer Analysis,” and “Triple Negative Breast Cancer.” Data were compared based upon log2 median-centered intensity in TCGA microarray datasets. Kaplan-Meier Plotter (http://kmplot.com/analysis/) was further used to assess the relationship between ADAMTS9-AS2 expression levels and TNBC patient survival outcomes.

### Microarray Analysis

Total RNA were isolated from the paired tissue samples of five TNBC patients using Trizol reagent kit (Invitrogen, Carlsbad, CA, USA) according to the manufacturer’s protocol. RNA quality was assessed on an Agilent 2100 Bioanalyzer (Agilent Technologies, Palo Alto, CA, USA) and checked using RNase free agarose gel electrophoresis. After total RNA was extracted, eukaryotic mRNA was enriched by Oligo(dT) beads. Second-strand cDNA were synthesized by DNA polymerase I, RNase H, dNTP and buffer. Then the cDNA fragments were purified with QiaQuick PCR extraction kit (Qiagen, Venlo, The Netherlands), end repaired, poly(A) added, and ligated to Illumina sequencing adapters. The ligation products were size selected by agarose gel electrophoresis, PCR amplified, and sequenced using Illumina Novaseq6000 by Gene Denovo Biotechnology Co. (Guangzhou, China).

### Statistical Analysis

Statistical analyses were performed by SPSS 20.0 (IBM, SPSS, USA) and GraphPad Prism 8.0 (GraphPad Software Inc., USA). Data were showed as mean ± standard deviation (SD). The differences between groups were assessed by Student’s t test, one-way ANOVA or χ2 test. Differences were considered statistically significant when P < 0.05.

## Results

### ADAMTS9‐AS2 Is Downregulated and Associated With Poor Prognosis in TNBC Patients

**Figure 1:**
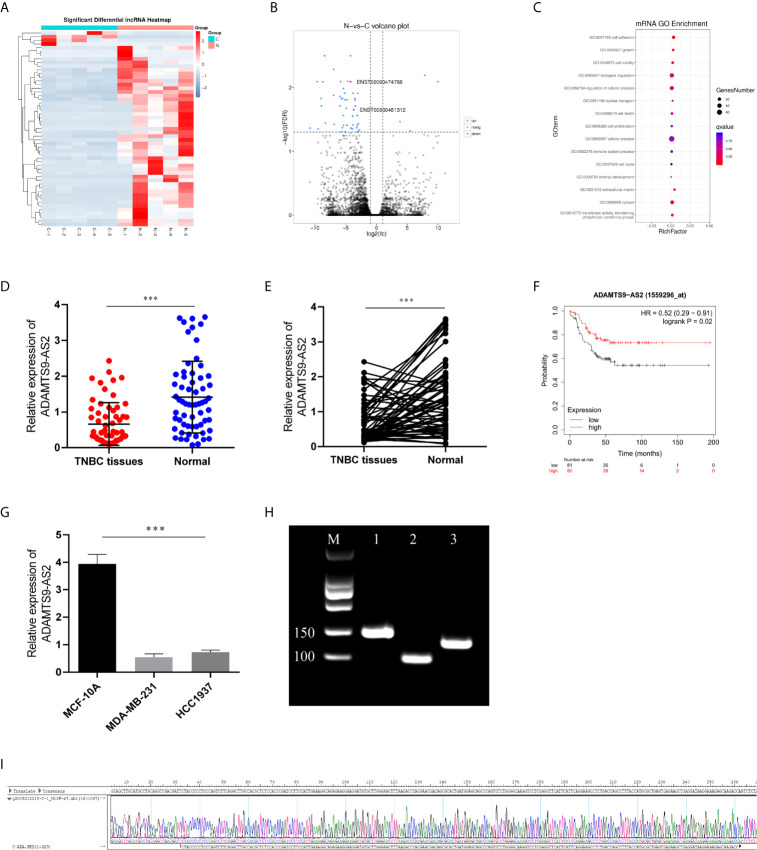
ADAMTS9‐AS2 is downregulated and associated with poor prognosis in TNBC patients. **(A, B)** The cluster heat maps and the volcano plot visualize the expression of lncRNA between TNBC tissues and adjacent non-tumor tissues. The red dots and green dots represent upregulated and downregulated LncRNAs with statistical significance, respectively. **(C)** GO pathway analysis of TNBC tissues. **(D, E)** Relative expression of ADAMTS9‐AS2 in 62 TNBC tissues and adjacent normal tissues. **(F)** Kaplan–Meier survival curve of patients with TNBC downloaded from TCGA database. **(G)** Relative expression of ADAMTS9‐AS2 in TNBC cell lines and normal breast cell line. **(H, I)** PCR product in agarose gel electrophoresis and the splicing site verified by DNA sequencing. ***P < 0.001.

We began by screening the most different lncRNAs and GO pathways that were differentially expressed (FC > 1.5, P < 0.01) in TNBC tissues relative to matched healthy control samples (**Figures 1A–C**). This analysis revealed significant ADAMTS9-AS2 downregulation in TNBC tumor tissues, and such downregulation was confirmed *via* qPCR (**Figures 1D, E**). Such ADAMTS9-AS2 downregulation was correlated with decreased TNBC patient survival (**Figure 1F**), and with TNM stage, age and tumor size (**Table 1**). Consistent with these patient findings, ADAMTS9-AS2 was downregulated in the MDA-MB-231 and HCC1937 TNBC cell lines relative to normal MCF-10A control cells (**Figure 1G**). The results of these qPCR analyses were validated *via* agarose gel electrophoresis and sequencing, revealing the presence of a single 104 bp product consistent with the length of the predicted sequence of this lncRNA (**Figures 1H, I**). As such, the downregulation of ADAMTS9-AS2 may play an important role in TNBC progression.

**Table 1:** Associations between ADAMS9-AS2 expression and clinicopathological parameters.

Clinicopathological parameters	Case No.	ADAMTS9-AS2		*P* value
		Low	High	
Total	62	30	32	
Age, yr				0.003**
<50	29	8	21	
≥50	33	22	11	
Tumor diameter, mm				0.011*
≤20	13	2	11	
>20	49	28	21	
Tumor stage(TNM)				0.04*
I	15	3	12	
II	36	21	15	
III	11	6	5	
Histological grade				0.876
I	14	5	9	
II	26	11	15	
III	22	14	8	
Lymph node metastasis				0.423
negative	41	18	23	
positive	21	12	9	
Ki67				0.102
low	19	6	13	
high	43	24	19	

Statistical analysis were carried out using Person χ^2^ test.

*P < 0.05, **P < 0.01 have statistical significances.

### ADAMTS9‐AS2 Suppresses TNBC Cell Proliferation

**Figure 2:**
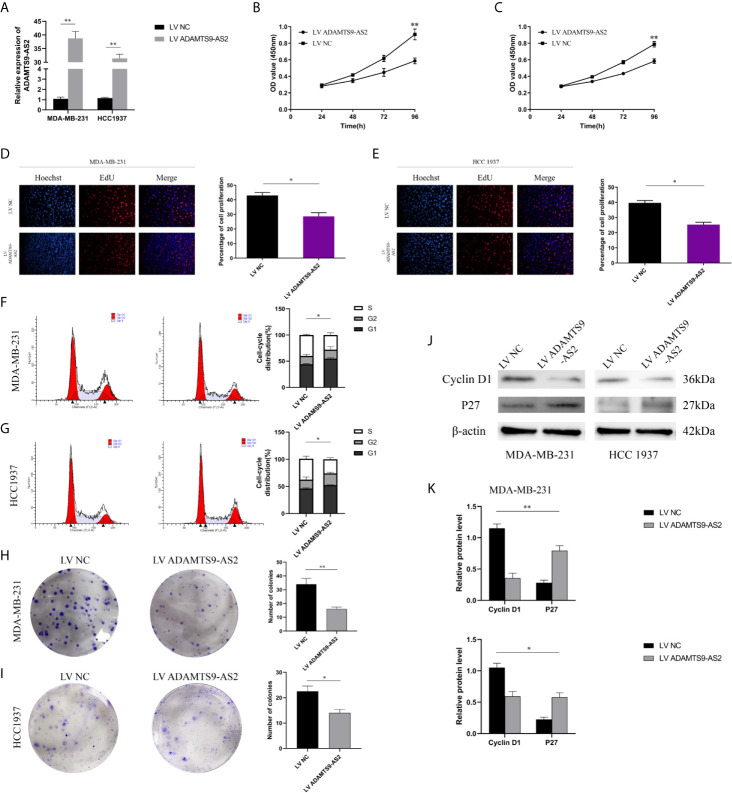
ADAMTS9‐AS2 suppresses TNBC cell proliferation. a: qRT-PCR analysis of LncRNA ADAMTS9-AS2 expression in TNBC cells transfected with ADAMTS9-AS2 overexpression vector or NC. **(B, C)** Growth curves of cells transfected with indicated vectors were evaluated by CCK8 assays. **(D, E)** EdU assays were conducted in cells after transfection with LV ADAMTS9-AS2 or LV NC. **(F, G)** The cell cycle progression was analyzed by flow cytometry after indicated transfected. **(H, I)** Colony formation assays were executed to detect the proliferation of TNBC cells transfected with indicated vectors. **(J, K)** Western blot was used to detect the influence of ADAMTS9-AS2 on cell cycle markers. *P < 0.05, **P < 0.01.

The functional role of ADAMTS9-AS2 was next evaluated by utilizing pcDNA vectors to overexpress this lncRNA in MDA-MB-231 and HCC1937 cells (**Figure 2A**). CCK-8 assays revealed that ADAMTS9-AS2 overexpression decreased the viability of these two BC cell lines, and EdU uptake assays confirmed this finding (**Figures 2B–E**). A cell cycle analysis subsequently revealed that ADAMTS9-AS2 overexpression increased the frequency of TNBC cells in the G0-G1 phase and reduced the frequency of cells in the S phase, suggesting that this lncRNA induced G1 phase arrest in these cancer cells (**Figures 2F, G**). A colony formation assay further revealed that ADAMTS9-AS2 overexpression markedly impaired colony formation (**Figures 2H, I**). Consistent with the cell cycle influence, the expression levels of cell cycle proteins were markedly changed when ADAMTS9-AS2 was enhanced (**Figures 2J, K**). As such, ADAMTS9-AS2 can suppress TNBC cell proliferation.

### ADAMTS9‐AS2 Regulates TNBC Cell Invasion, Metastasis, and Cell Cycle Progression and Inhibited Warburg Effect

**Figure 3:**
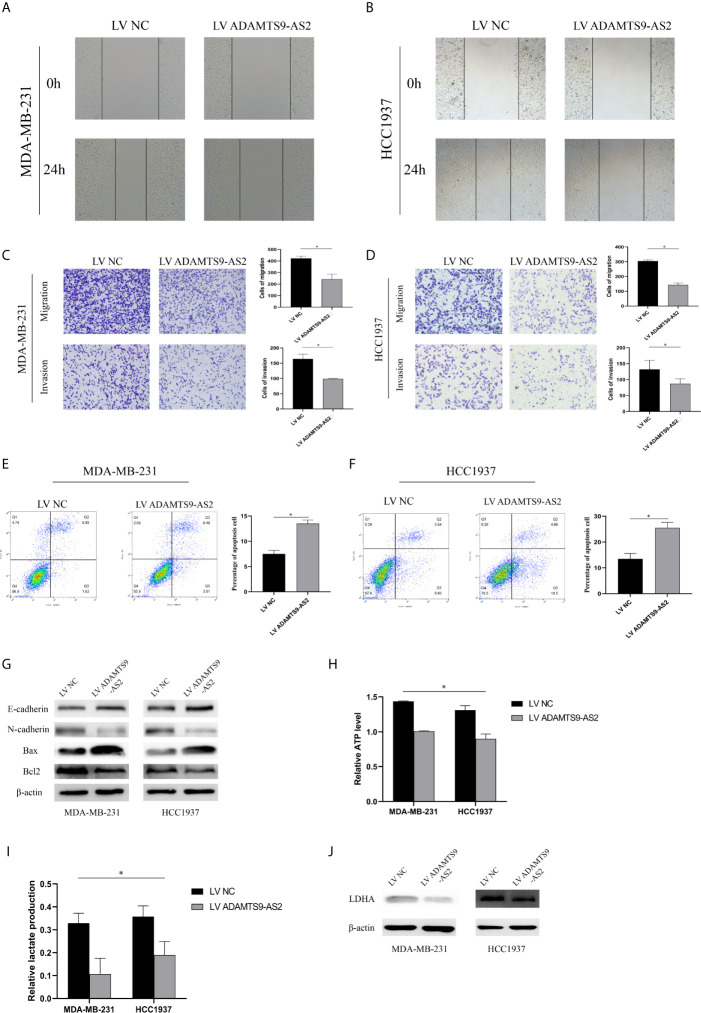
ADAMTS9‐AS2 regulates TNBC cell invasion, metastasis, and cell cycle progression and inhibited Warburg effect. **(A, B)** Cell migration capacities were detected by wound healing assays after transfected with indicated vectors. **(C, D)** Cell migration and invasion abilities were determined by transwell assays after transfection. **(E, F)** Apoptosis rate of TNBC cells was analyzed by flow cytometry after LV ADAMTS9-AS2. **(G)** The expression levels of apoptosis-related and epithelial-mesenchymal transition process marker proteins were determined by western blot. **(H, I)** Production of lactate and ATP were examined in TNBC cells transfected with LV ADAMTS9-AS2 or LV NC as indicated. **(J)** related LDHA expression was detected by western blot. *P < 0.05.

**Figure 4:**
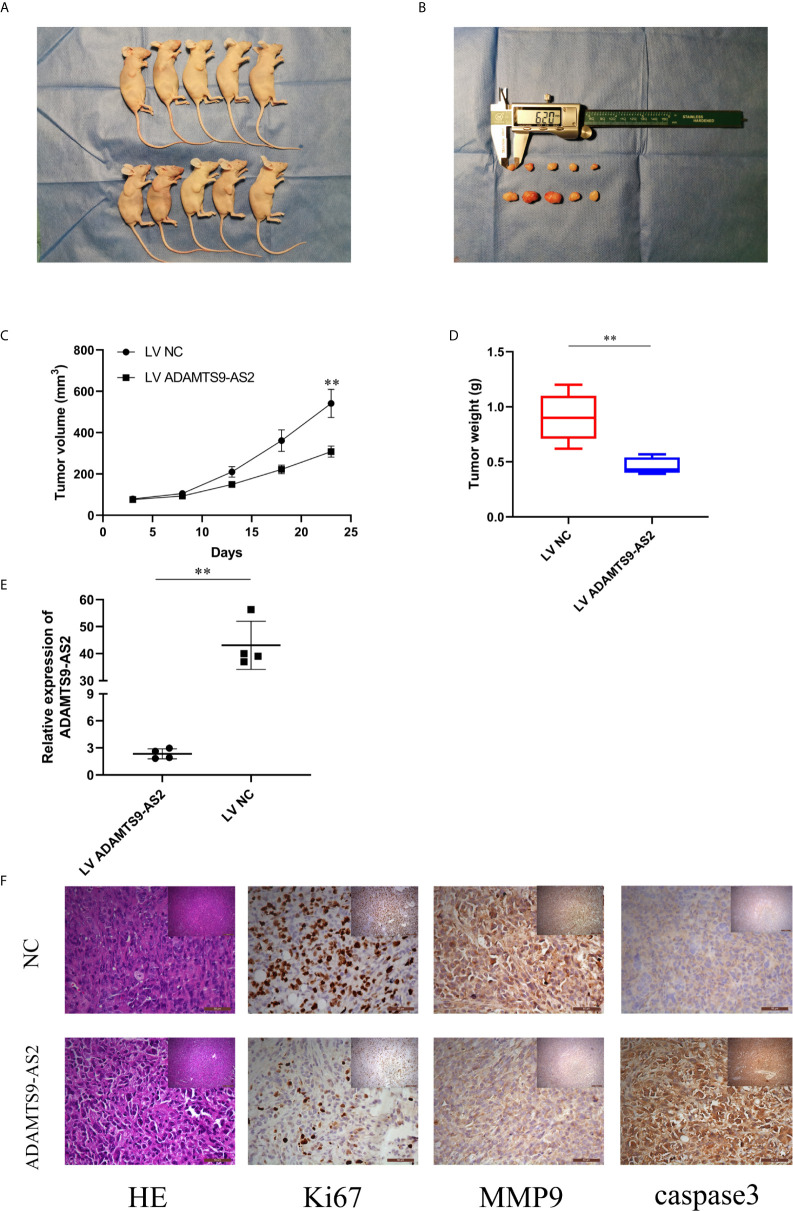
Overexpression of ADAMTS9‐AS2 suppresses *in vivo* BC tumor growth. **(A, B)** Representative images of xenograft tumors of each group. **(C)** Growth curves of xenograft tumors which were measured every 5 days. **(D)** Tumor weights from two groups are represented. **(E)** qRT-PCR detected relative expression in two groups. **(F)** IHC staining was applied to analyze the protein levels of Ki67, MMP9 and cleaved-caspase 3. **P < 0.01.

The role of ADAMTS9-AS2 as a regulator of TNBC cell invasion was next evaluated through Transwell and wound healing assays, which demonstrated that the upregulation of this lncRNA impaired both of these activities in MDA-MB-231 and HCC1937 cells (**Figures 3A–D**). Flow cytometry analyses further revealed that MDA-MB-231 and HCC1937 cells overexpressing ADAMTS9-AS2 exhibited higher rates of apoptotic cell death relative to cells transfected with negative control constructs (**Figures 3E, F**). Western blotting also revealed that ADAMTS9-AS2 overexpression increased the levels of Bax in these tumor cells, whereas such overexpression suppressed Bcl-2 expression and EMT-related protein relative to control cells (**Figure 4G**). The glycolytic pathway is the main metabolic pathway for tumor cells to perform energy metabolism. In this process, every molecule of glucose taken by cancer cells can quickly generate 2 molecules of ATP to meet their own energy needs. ADAMTS9-AS2 decreased the production of lactate and ATP in MDA-MB-231 cells (**Figures 3H, I**). Since LDHA is a marker of glycolytic pathway, we detected the expression of LDHA in TNBC after transfection and ADAMTS9-AS2 inhibited the expression of LDHA (**Figure 3J**). As such, ADAMTS9-AS2 may suppress TNBC progression *in vitro.*

### Overexpression of ADAMTS9‐AS2 Suppresses *In Vivo* BC Tumor Growth

To evaluate the ability of ADAMTS9-AS2 to impact TNBC progression *in vivo*, we next transduced MDA-MB-231 cells with LV-NC or LV-ADAMTS9-AS2 and then implanted these tumor cells subcutaneously in mice. Tumors overexpressing ADAMTS9-AS2 were smaller than those transduced with the control lentivirus (**Figures 4A–C**), and these tumors weighed significantly less than control tumors (**Figure 4D**). As expected, ADAMTS9-AS2 was expressed at a higher level in tumors from the overexpression group relative to the NC group (**Figure 4E**). Subsequent immunohistochemical staining revealed that Ki-67 AND MMP9 expression levels were decreased in tumors overexpressing ADAMTS9-AS2 (**Figure 4F**). Together, these findings thus indicated that ADAMTS9-AS2 can suppress *in vivo* tumor growth.

### ADAMTS9-AS2 Interacts With RPL22 in TNBC Cells to Suppress Tumor Progression

**Figure 5:**
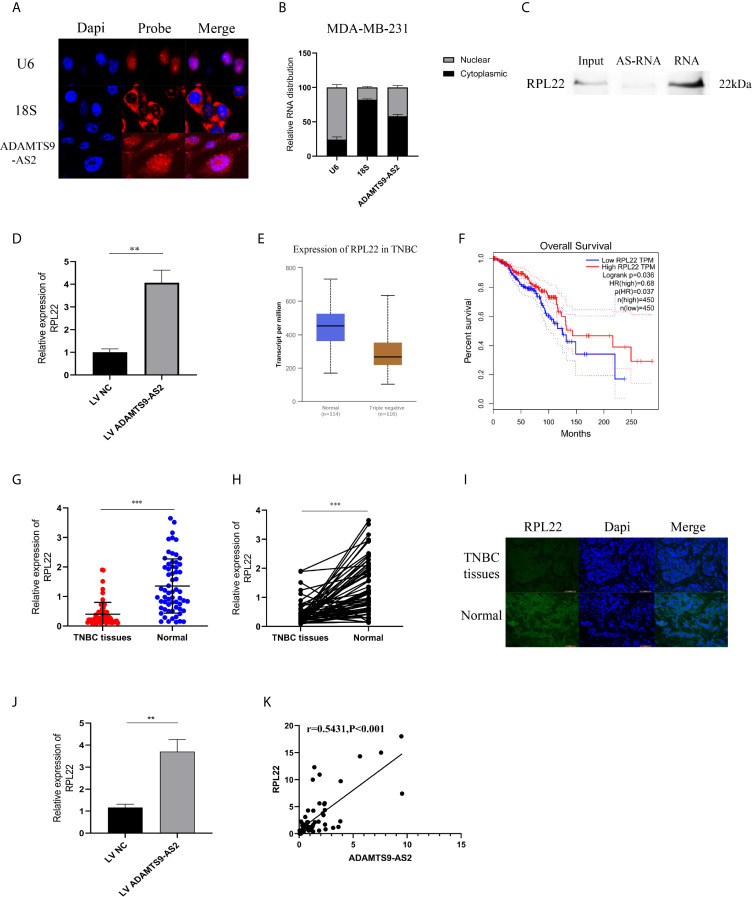
ADAMTS9-AS2 interacts with RPL22 in TNBC cells to suppress tumor progression. **(A)** FISH analysis of the location of ADAMTS9-AS2 in the cytoplasm and nuclear fractions of MDA-MB-231 cells. **(B)** qRT-PCR was used to detect ADAMTS9-AS2 subcellular fractionation. **(C)** RNA pull-down assay indicated that ADAMTS9-AS2 interacted with RPL22. **(D)** Western blot proved the relationship between ADAMTS9-AS2 interacted with RPL22. **(E)** Relative expression of RPL22 in TNBC tissues (Tumor) compared with normal tissue (normal) was analyzed using TCGA data. **(F)** Kaplan-Meier survival analysis of overall survival based on TCGA data. **(G)** Relative expression of RPL22 in TNBC tissues (Tumor) and adjacent non-tumor tissues (Normal) was determined by qRT-PCR (n = 62). **(I)** IF was used to demonstrate the relative expression of RPL22 in TNBC tissues. **(J)** Relative expression of RPL22 in xenograft tumors of each group was determined by qRT-PCR. **(K)** Spearman–Pearson correlation of ADAMTS9-AS2 and RPL22. **P < 0.01, ***P < 0.001.

FISH and subcellular localization analyses were next confirmed to evaluate the localization of ADAMTS9-AS2 in TNBC cells, revealing it to be present within both the cytoplasm and nucleus (**Figures 5A, B**). Given that we were able to detect this lncRNA in the nucleus, we next utilized biotinylated ADAMTS9-AS2 to conduct a pull-down assay using MDA-MB-231 cell lysates, which were then separated *via* SDS-PAGE. Subsequent mass spectrometry (MS) analyses led to the identification of RPL22, which is a key ribosomal protein (RP) associated with ribosome biogenesis and protein translation, as being specifically present within the ADAMTS9-AS2 pulldown lane. This pulldown assay thus confirmed the ability of ADAMTS9-AS2 and RPL22 to interact with one another (**Figure 5C**). RPL22 mRNA and protein levels were significantly increased in TNBC following ADAMTS9-AS2 overexpression (**Figure 5D**). In the TCGA database, RPL22 was shown to be expressed at lower levels in tumor relative to control breast tissue samples, with such downregulation being correlated with poorer prognosis (**Figures 5E, F**). When we assessed RPL22 levels *via* Immunofluorescence of TNBC patient tumor and control tissues and *via* qPCR in 62 TNBC tissue samples, we confirmed that this RP was present at lower levels in tumor tissues **(Figures 5G–I)**. qRT-PCR further confirmed higher levels of RPL22 expression in xenograft tumors overexpressing ADAMTS9-AS2 (**Figure 5J**). ADAMTS9-AS2 was also positively correlated with RPL22 in TNBC tissues (*r* = 0.5431, *P* < 0.001, **Figure 5K**).

### ADAMTS9-AS2 Regulates RPL22 to Control TNBC Progression Through RPL22

**Figure 6:**
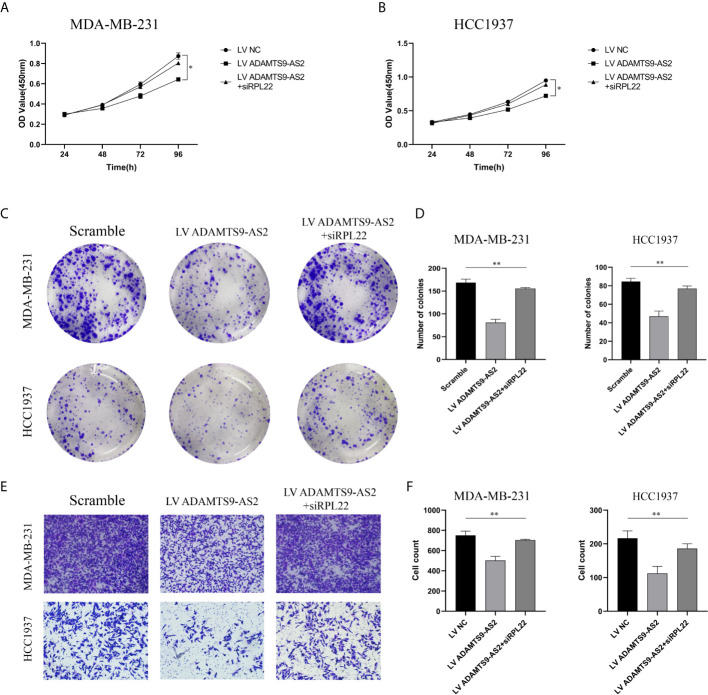
ADAMTS9-AS2 regulates RPL22 to control TNBC progression through RPL22. **(A–D)** The cell proliferation was determined after transfection with LV ADAMTS9-AS2, ADAMTS9-AS2+siRPL22 and LV NC by CCK-8 and Colony formation assay. **(E, F)** The cell migration was determined after transfection by Transwell assay. *P < 0.05, **P < 0.01.

We next conducted rescue experiments to confirm whether RPL22 was involved in ADAMTS9-AS2-mediated suppression of TNBC tumor growth. Through CCK-8 and colony formation assays, we confirmed that ADAMTS9-AS2 overexpression impaired the viability of these cells, whereas knocking down RPL22 reversed this effect (**Figures 6A–D**). Similarly, Transwell assays revealed that ADAMTS9-AS2 overexpression was able to suppress the migrative activity of TNBC cells, while si-RPL22 co-transfection reversed these phenotypic changes (**Figures 6E, F**).

### ADAMTS9-AS2 Regulates the TGF-β Signaling Pathway to Control TNBC Progression

**Figure 7:**
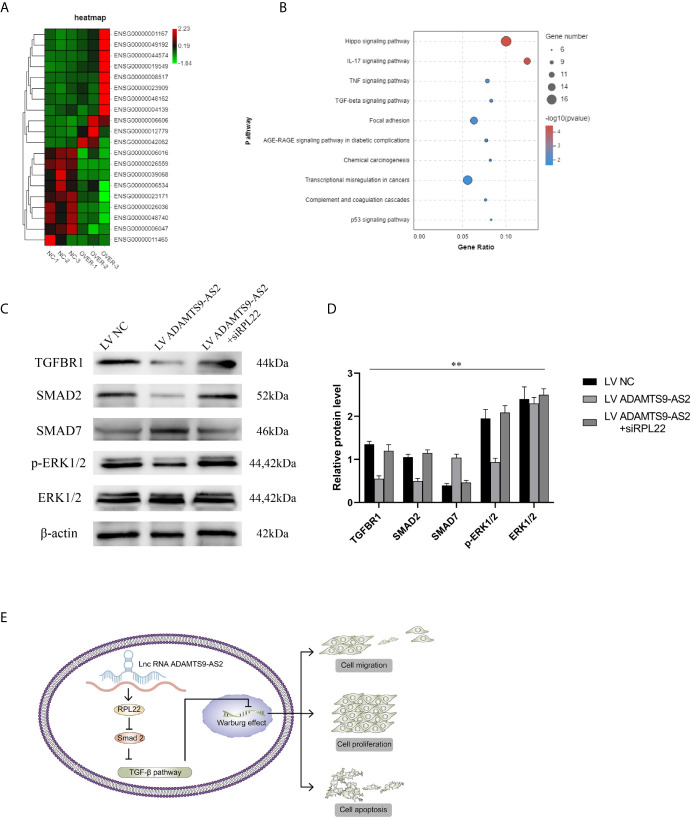
ADAMTS9-AS2 regulates the TGF-β signaling pathway to control TNBC progression. **(A)** The cluster heat maps displayed the 20 most representative differentially expressed mRNAs with transcriptomic sequencing. **(B)** KEGG pathway analysis of differentially expressed mRNAs. **(C, D)** Western blot assay determined the total and active protein level of TGF-β signaling pathway related proteins in MDA-MB231 cell lines. **(E)** Summary of the mechanism of ADAMTS9-AS2 in TNBC cell lines. **P < 0.01.

Lastly, we performed RNA-seq analyses of MDA-MB-231 cells overexpressing ADAMTS9-AS2 to further explore its functional role in this oncogenic context. Heatmap and KEGG analyses revealed that ADAMTS9-AS2 overexpression impacted the TGF-β signaling pathway (**Figures 7A, B**). Given previously reported interactions between RPL22 and Smad2 (20), we next assessed the ability of RPL22 to suppress TGF-β signaling. Following ADAMTS9-AS2, siRPL22, and RPL22 transfection into TNBC cell lines, we evaluated changes in TGF-β-related gene expression. Western blotting revealed that ADAMTS9-AS2 and RPL22 controlled TGFBRI, smad2, smad7, and p-ERK1/2 protein levels (**Figures 7C, D**). Together, these findings suggest that ADAMTS9-AS2 controls the survival, proliferation, and malignancy of TNBC cells *via* the TGF-β pathway (**Figure 7E**).

## Discussion

The advent of next-generation sequencing has led to the identification of many differentially regulated lncRNAs in TNBC (21), some of which have been identified as key diagnostic or prognostic biomarkers of this disease type (22). For example, the M2 macrophage-induced lncRNA PCAT6 regulates VEGFR2 expression and thereby controls TNBC growth and angiogenesis (23). Li. et al. found that the MNX1-AS1 lncRNA was able to enhance STAT3 phosphorylation, thereby driving TNBC progression (24). ADAMTS9-AS2 has also previously been shown to suppress lung and ovarian cancer growth (17, 19), but its functional role in TNBC has not been previously clarified.

Herein, we established that ADAMTS9-AS2 is an important regulator of TNBC. RNA sequencing and subsequent bioinformatics analyses identified ADAMTS9-AS2 as being among the lncRNAs that were most significantly downregulated in TNBC tumor samples relative to matched paracancerous tissue samples. The downregulation of this lncRNA was associated with patient age, tumor size, lymph node status, higher TNM stage, and a poorer prognosis. When functional experiments were conducted, we determined that ADAMTS9-AS2 was able to suppress the proliferative and invasive activity of TNBC cells *in vitro* and *in vivo*, instead promoting their apoptotic death. There are multiple mechanisms whereby lncRNAs can modulate cellular physiology, including by sequestering miRNAs (25), controlling protein stability (26), or regulating chromatin structure (27). ADAMTS9-AS2 is an antisense transcript derived from the ADAMTS9 protein-coding gene. Such antisense RNAs have been shown to function as post-transcriptional regulators of RNA and protein stability and of promoter activation. As we found that ADAMTS9-AS2 was largely localized within BC cell nuclei, this suggested that it may influence the biology of these cells by interacting with specific proteins. Pull-down assays revealed that ADAMTS9-AS2 was able to regulate RPL22 expression, and this regulatory relationship was confirmed *via* qPCR and Western blotting.

Ribosomes contain essential RPs that are necessary for facilitating translation in the cytoplasm. RPs are important RNA-binding proteins that are present at high levels in all cell types (28). Altered ribosomal biogenesis and protein translation can profoundly shape tumor progression. BC tumors exhibit the upregulation of RPL19 (29), whereas RPL7A is downregulated in osteosarcoma (30), and RPL41 is downregulated in BC (31). Such changes in RP expression can alter tumor cell malignancy by modulating their proliferative activity (28). However, relatively little is known about the prognostic relevance of specific RPs in BC. Herein, we found that BC tumors exhibit significant RPL22 downregulation as compared to healthy tissue samples at the mRNA and protein levels. RPL22 is a 60S large ribosomal subunit component, and mutations in this gene have been linked to bacterial macrolide resistance (32). This RP has also been linked to viral infections, protecting against EBV-infected cell transformation (33), and controlling the development of T cells by controlling apoptotic cell death (34). RPL22 mutations may enhance cellular proliferation (35) and promote oncogenesis. Herein, we found that RPL22 expression to be positively correlated with that of ADAMTS9-AS2, and rescue experiments confirmed the ability of ADAMTS9-AS2 to regulate TNBC cell progression. In prior reports, RPL22 was shown to control TGF-βpathway activation (36), with TGF-β signaling being closely linked to cancer progression and metastasis (37, 38). Among them, TGFBR, SMAD2 and SMAD7 were the most known regulators, so we examined whether ADAMTS9-AS2 could affect their expression. In the KEGG map, the TGF-βinduced the activation of MAPK/ERK pathway, which promote cancer progression in TNBC, so we detected relative protein and found the possible mechanism ADAMTS9-AS2 may involve in.

In brief, we have discussed the function and mechanism of ADAMTS9-AS2. However, some questions remained for further study. Firstly, the clinical samples are not enough. As the expression was associated with age, we wondered whether there is any relationship between cell aging and ADAMTS9-AS2, resulting in a new therapeutic target for elderly TNBC patients. What’s more, we could see a tendency between the LncRNA and lymph node metastasis and Ki67, making our theory more persuasive. Then, based on our experiment, about 40% of the LncRNA was located in cytoplasm, whether ADAMTS9-AS2 could play the anti-tumor role through the cytoplasmic LncRNA remained to be studied.

As such, we hypothesized that the downregulated ADAMTS9-AS2 levels were associated with a poorer TNBC patient prognosis. ADAMTS9-AS2 could regulate TNBC cells proliferation, apoptosis, metastasis and Warburg effect by ADAMTS9-AS2/RPL22 axis *via* modulation of the TGF-β pathway. We identified ADAMTS9-AS2 as a potential therapeutic target in patients with TNBC, although further studies will be needed to validate this possibility.

## Data Availability Statement

The original contributions presented in the study are included in the article/**Supplementary Material**, further inquiries can be directed to the corresponding authors.

## Ethics Statement

The studies involving human participants were reviewed and approved by Affiliated Hospital of Nantong University. The patients/participants provided their written informed consent to participate in this study. The animal study was reviewed and approved by Affiliated Hospital of Nantong University. Written informed consent was obtained from the individual(s) for the publication of any potentially identifiable images or data included in this article.

## Author Contributions

All authors listed have made a substantial, direct, and intellectual contribution to the work, and approved it for publication.

## Funding

This study was partially supported by a Nantong City-level Science and Technology Plan Project fund from KN. (Number GJZ17086.)

## Conflict of Interest

The authors declare that the research was conducted in the absence of any commercial or financial relationships that could be construed as a potential conflict of interest.
